# Identifying protein complexes based on an edge weight algorithm and core-attachment structure

**DOI:** 10.1186/s12859-019-3007-y

**Published:** 2019-09-14

**Authors:** Rongquan Wang, Guixia Liu, Caixia Wang

**Affiliations:** 1 College of Computer Science and Technology, Jilin University, No. 2699 Qianjin Street, Changchun, 130012 China; 2 Key Laboratory of Symbolic Computation and Knowledge Engineering of Ministry of Education, Jilin University, No. 2699 Qianjin Street, Changchun, 130012 China; 3 School of International Economics, China Foreign Affairs University, 24 Zhanlanguan Road, Xicheng District, Beijing, 100037 China

**Keywords:** Protein complexes, Protein-protein interaction networks, Core-attachment structure, Spurious interactions, Structural similarity

## Abstract

**Background:**

Protein complex identification from protein-protein interaction (PPI) networks is crucial for understanding cellular organization principles and functional mechanisms. In recent decades, numerous computational methods have been proposed to identify protein complexes. However, most of the current state-of-the-art studies still have some challenges to resolve, including their high false-positives rates, incapability of identifying overlapping complexes, lack of consideration for the inherent organization within protein complexes, and absence of some biological attachment proteins.

**Results:**

In this paper, to overcome these limitations, we present a protein complex identification method based on an edge weight method and core-attachment structure (EWCA) which consists of a complex core and some sparse attachment proteins. First, we propose a new weighting method to assess the reliability of interactions. Second, we identify protein complex cores by using the structural similarity between a seed and its direct neighbors. Third, we introduce a new method to detect attachment proteins that is able to distinguish and identify peripheral proteins and overlapping proteins. Finally, we bind attachment proteins to their corresponding complex cores to form protein complexes and discard redundant protein complexes. The experimental results indicate that EWCA outperforms existing state-of-the-art methods in terms of both accuracy and *p*-value. Furthermore, EWCA could identify many more protein complexes with statistical significance. Additionally, EWCA could have better balance accuracy and efficiency than some state-of-the-art methods with high accuracy.

**Conclusions:**

In summary, EWCA has better performance for protein complex identification by a comprehensive comparison with twelve algorithms in terms of different evaluation metrics. The datasets and software are freely available for academic research at https://github.com/RongquanWang/EWCA.

## Background

A significant task of system biology is to explore cellular function and organization by studying the PPI networks. Most of the functional processes within a cell are executed by protein complexes [1]. Therefore, the identification of protein complexes is an important research problem in systems biology. In addition, understanding the biological functions is a fundamental task for different cellular systems and is beneficial for treating complex diseases. Due to the development of advanced high-throughput techniques, a large number of PPI networks have been generated [2], which makes discovering protein complexes more convenient. However, how to accurately identify biological protein complexes has been an important research topic in the post-genomic era [3]. The accurate identification of protein complexes in PPI networks is significant for understanding the principles of cellular organization and function [4]. As a result, a large number of methods including laboratory-based and computational-based methods have been proposed to address this issue.

So far, some biologically experimental methods have been proposed to detect protein complexes from the PPI networks. However, these methods require high level of expensive cost and time-consuming. Thus, many efficient alternative computational methods are proposed to identify protein complexes in PPI networks. Moreover, a number of high-quality and large-scale PPI networks provide possible for computational methods to identify protein complexes. Generally, a PPI network can be modeled as an undirected graph (also called a network), where vertices represent proteins and edges represent interactions between proteins. Various state-of-the-art computational methods have been developed to identify protein complexes in the last few years. According to the use of information in identifying process, these computational methods are classified into two categories. One category only uses the topological information of PPI networks to identify protein complexes, and we call them topology-based methods. The other category is to combine the biological and topological information to identify protein complexes, such as IPC-BSS [5], GMFTP [6] and DPC [7], etc.

A large amount of topology-based methods have been proposed to identify protein complexes by employing different topological structures. For instance, CFinder [8] and CMC [9] are based on cliques or k-cliques; MCL [10], DPClus [11] and SPICi [12] use dense subgraph; ClusterONE [13] and CALM [14] depend on modularity concept; Core [15] and COACH [16] employ core-attachment structure. Moreover, ProRank+ [17] uses a ranking algorithm and spoke model for identifying protein complexes. All above methods are typical topology-based methods. Up to now, there is no clear and appropriate definition states that a group of proteins should be in the same complex in a PPI network.

As we all known, a clique is a complete subgraph and its all vertices are connected to each other. Some researchers believe that cliques or k-cliques are protein complexes. For example, CFinder [8] is based on clique percolation method (CPM) [18] which identifies the k-cliques. However, it is too strict to require a protein complex being a clique or k-clique, and it is computationally infeasible in the larger PPI networks, because it is NP-Complete [19]. Furthermore, many studies assume that dense subgraph corresponds to protein complex. The reason is that proteins in the same protein complex interact frequently among themselves [20, 21]. MCL [10] is highly scalable clustering algorithm based on simulating random walk in biological networks. Another example is a fast heuristic graph clustering method, which is called SPICi [12], which selects the highest weighted node as a seed, and it is expanded according to local density and support measure. SPICi is efficiency methods for identifying protein complexes. However, it has low accuracy and can not identify overlapping protein complexes. In fact, some protein complexes are usually overlapping and many multi-functional proteins are involved in different protein complexes.

Consequently, some new computational methods have been proposed to identify overlapping protein complexes. For example, DPClus [11] is a seed-growth method based on different graph topological characteristics such as degree, diameter, density and so on. The main differences among them are density threshold and cluster expanding strategy [22]. More importantly, they may miss some low dense protein complexes [14]. Moreover, there are 408 known *yeast* protein complexes which are provided by Pu et al. in [23], 21% complexes’ density is lower than 0.5. Additionally, there exists high false-positive interactions in the PPI networks. Therefore, some methods try to assess the reliability of existing PPIs and filter out the unreliable interactions [24] such as PEWCC [25] and ProRank+ [17]. All of these methods are based on the single topological structure of protein complexes and do not utilize the information of known protein complexes.

Furthermore, some researchers find that many protein complexes have modularity structure, which means these protein complexes are densely connected within themselves but sparsely connected with the rest of the PPI networks [21, 26–28]. Motivated by this issue, a number of new clustering methods based on modularity structure have been proposed, including ClusterONE [13], CALM [14], EPOF [29] and PCR-FR [30], etc. One of most widely known is ClusterONE [13]. ClusterONE can identify overlapping protein complexes from the PPI networks, and authors introduce the maximum matching ratio (MMR) to evaluate predicted overlapping protein complexes. However, ClusterONE may neglect the effect of overlapping proteins in the process of identifying seeds [14] and some attachment proteins may be missed [28].

Recently, some research results have shown that the characteristics of detected protein complexes indicate that protein complexes generally have a core-attachment structure [31–34]. Gavin et al. [31] have revealed that proteins within a protein complex are organized as core proteins and attachment proteins. Although there is no detailed statement for this structure, some researchers think that a protein complex core is often a dense subgraph and that some attachment proteins are closely associated with its core proteins and assist these core proteins to perform subordinate functions [16]; then, together they form a biologically meaningful protein complex. Ahmed et al.’s studies also demonstrate a similar architecture and inherent organization in protein complexes [15, 33, 35].

Up to now, several methods based on core-attachment structure have been explored for identifying protein complexes, such as COACH [16], Core [15] and Ma et al.’s method [22]. These methods have a good performance dramatically, and demonstrate the significance of this structure [22]. Methods based on core-attachment structure are generally divided into two stages. In identifying complex cores phase, they are mainly to identify dense subgraph or maximal clique as protein complex core. In fact, some protein complex cores are dense subgraph or maximal clique, but other are not high-density [23]. Ma et al. [22] have argued that the density of a subgraph is not appropriate to characterize a protein complex core. In identifying attachment proteins phase, most of methods based on core-attachment structure follow Wu et al.’ criterion [16] that is to select the proteins whose neighbors interact with more than half of the proteins in its protein complex core. As we know the PPI networks are sparse and have proved that the size of protein complex cores varies from 1 to 23 [31]. Obviously, it could be sufficient to describe the relation between a protein complex core and their attachment proteins. However, the currently available PPI networks contain many false-positives interactions which greatly affect protein complexes detection accurately.

**Fig. 1:**
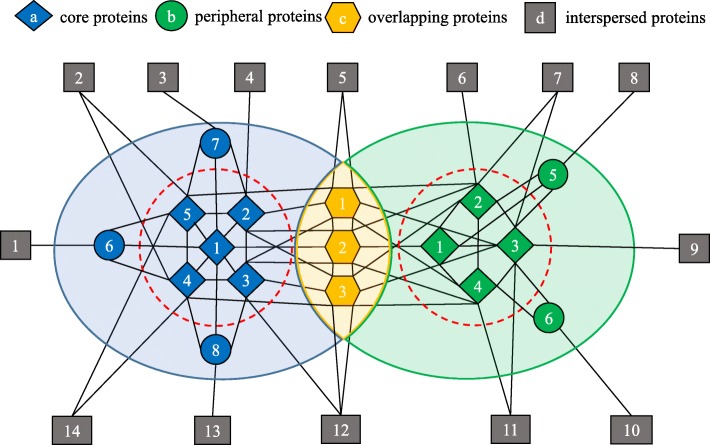
A network with two protein complexes and three overlapping proteins. Each protein complex consists of core proteins, peripheral proteins and three overlapping proteins which are shared by two protein complexes in overlapping yellow area. Additionally, these core proteins inside the red dotted circle constitute their protein complex cores. Note that diamond nodes present core proteins, circle nodes present peripheral proteins, hexagonal nodes present overlapping proteins, parall elogram nodes present interspersed proteins

In this paper, we try to overcome these limitations and employ a protein complex internal structure to identify biologically and accurately meaningful protein complexes. Inspired by some reserachers’s [14, 32, 36–38] experimental works and the distinctive properties of core and attachment proteins. We further study the core-attachment structure. However, these previous studies only illustrate some concepts of this structure but do not give a method for how to identify various types of proteins including core proteins, peripheral proteins and overlapping proteins [14]. In real PPI networks, the overlapping protein complexes are universal [14]. Therefore, the overlapping proteins often play an important role in the identification of protein complexes. Generally, overlapping proteins are regard as member of two or more protein complexes at the same time. The overlapping proteins promote the interaction between protein complexes. In addition, in many real complex networks, the identification of overlapping nodes is useful in the social network, cited network, world wide web and so on. Most of the algorithms we mentioned before do not have the ability to differentiate and identify overlapping proteins and peripheral proteins while we extend the ability of EWCA. Thus, in this paper, we provide some definitions to distinguish and identify local overlapping proteins and locally peripheral proteins, which has not been done by other researchers. We take a simple example to show core-attachment structure in Fig. 1. We propose a method which is named EWCA, to identify protein complexes. Most existing protein complex identification approaches search for protein complexes based on ’density graph’ assumptions. Unlike some of them, EWCA provides a new direction to use a Core-attachment structure to identify protein complexes. First, EWCA defines a new edge weight measure to weight and filter out interactions in PPI networks. Second, EWCA could generate some preliminary overlapping complex cores based on structural similarity rather than density. This approach is more reasonable because the core proteins in the same complex core have relatively more structural similarity. Third, EWCA designs a new method to discover attachment proteins for corresponding to the complex core. Finally, the experimental results show that EWCA performs better than existing state-of-the-art methods in terms of some evaluation metrics (e.g., F-measure and MMR) and functional enrichment.

## Preliminary

Generally, a PPI network can be typically modeled as an undirected graph *G*_*ppi*_=(*V*_*ppi*_,*E*_*ppi*_), where *V*_*ppi*_ represents as the set of vertices corresponding to proteins and *E*_*ppi*_ stands for the set of edges which represent the interactions between proteins from *V*_*ppi*_. A PPI network is undirected and may be unweighted or weighted, with weight on an edge representing the confidence score (usually between 0 and 1) for an interaction. For a vertex *v*, *N*(*v*) stands for the set of all vertex *v*’neighbors.

## Methods

### Construction of a reliable weighted PPI network

Generally speaking, the PPI networks obtained from different experimental methods are quite noisy (many interactions are believed to be false positives) [39]. Hence we should reduce the false positives. To address this challenge, some researchers have proposed preprocessing strategies to assess and eliminate potential false positives by using the topological properties of the PPI networks [40–43]. Meanwhile, some experimental results [44, 45] have shown that the PPIs with high confidence scores are assessed by the neighbor information-based methods, and these methods tend to be more reliable than others. Thus, we introduce a Jaccard’s coefficient similarity (JCS) measure proposed by Jaccard et al. [46]. The Jaccard’s coefficient similarity between two neighbor proteins *v* and *u* is defined by Eq. (1):

1 $$\begin{array}{@{}rcl@{}} JCS(v,u)= \left\{\begin{array}{ll} \frac{|CN(v,u)|}{|N(v)\cup N(u)|}, &|N(v)\cap N(u)| \geqslant 1, \cr 0, &otherwise, \end{array}\right. \end{array} $$

where *N*(*v*) and *N*(*u*) stand for the set of neighbor nodes of nodes *v* and *u*, respectively. *N*(*v*)∩*N*(*u*) is the set of all common neighbors between nodes *v* and *u*, and is denoted by *CN*(*v, u*). |*N*(*v*)∩*N*(*u*)| stands for the number of all common neighbors of *v* and *u*. |*N*(*v*)∪*N*(*u*)| represents the union set of all distinct neighbors of *v* and *u*. Obviously, the more common neighbors two proteins share, the higher similarity between two adjacent nodes. Here, to better quantify the connectivity between two adjacent nodes *v* and *u*, then we define a new high-order common neighbor (HOCN) similarity measure based on the Jaccard’s coefficient between node *v* and node *u*, and we introduce HOCN as follows. The main idea is to estimate each edge according to the common neighbors of the common neighbors of the two adjacent nodes. *HOCN*(*v, u*) is defined as Eq. (2):

2 $$ HOCN(v,u)=\frac{(JCS(v,u)+CNS(v,u))}{(|CN(v,u)|+1)},  $$

where

3 $$ CNS(v,u)=\sum_{w\in CN(v,w)}(JCS(v,w)*JCS(w,u)),  $$

The weight of the edge (v,u) between protein *v* and protein *u* is determined by not only the Jaccard’s coefficient between proteins *v* and *u* but also the probability that their common neighbors do support the edge (*v, u*). All common neighbors support (CNS) the edge (*v, u*) are calculated by Eq. (3). Finally, the weight of the edge (*v, u*) is determined by Eq. (2).

**Fig. 2:**
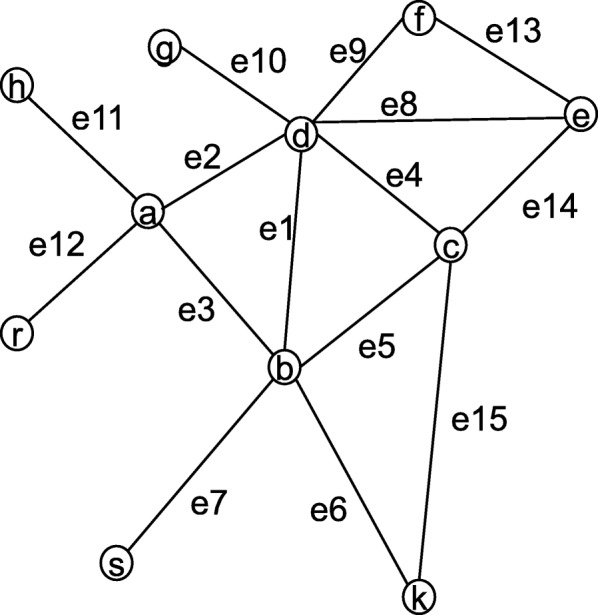
A simple hypothetical network of 11 proteins and 15 interactions which is used for illustrating how to determine the weight of the edge e1

To assess the reliability of protein interactions process, we give an example as shown in Fig. 2. Suppose we assess the weight of edge *e*1 between *b* and *d*. According to Eq. (1), we can obtain $JCS(b,d)=\frac {|\{a,c\}|}{|\{a,b,c,d,e,f,g,k,s\}|}= \frac {2}{9}$, $JCS(b,a)=\frac {|\{d\}|}{|\{a,b,c,d,k,h,r,s\}|}= \frac {1}{8},JCS(a,d)=\frac {|\{b\}|}{|\{a,b,c,d,e,f,g,h,r\}|}= \frac {1}{9},JCS(b,c)=\frac {|\{d,k\}|}{|\{a,b,c,d,e,k,s\}|}= \frac {2}{7},JCS(c,d)=\frac {|\{b,e\}|}{|\{a,b,c,d,e,f,g,k\}|}= \frac {2}{8}$. According to Eq. (3), the common proteins *a* and *c* support the edge *e*1 is $JCS(a,b)*JCS(a,d)=\frac {1}{8}*\frac {1}{9}=\frac {1}{72}$ and $JCS(b,c)*JCS(c,d)=\frac {2}{7}*\frac {2}{8}=\frac {4}{56}$, respectively. Therefore, the common proteins *a* and *c* support the edge *e*1 are *JCS*(*v, a*)∗*JCS*(*a, u*) + $JCS(v,c)*JCS(c,u)=\frac {1}{72}+\frac {4}{56}$. What’s more, the probability of edge *e*1 between proteins *d* and *b* is $JCS(d,b)=\frac {2}{9}$ based on Eq. (1). Finally, the weight of edge e1 is $\frac {\frac {2}{9}+\frac {1}{72}+\frac {4}{56}}{2+1}\approx 0.102$ according to Eq. (2).

Here, we use *HOCN*(*v, u*) to calculate the weight of each pair of edge (*v, u*) so that EWCA improves the quality of the identified protein complexes. Obviously, *HOCN*(*v, u*) considers more widely about the connectivity of the entire neighborhood of two adjacent nodes and may well determine whether two interactional proteins belong to the same protein complex. If $|N_{v} \cap N_{u}| \geqslant 1$, then *HOCN*(*v, u*) is the weight of edge (*v, u*). Otherwise, edge (*v, u*) is considered unreliable and it has to be discarded. The more details pseudo-codes of this phase is shown in Algorithm 1.


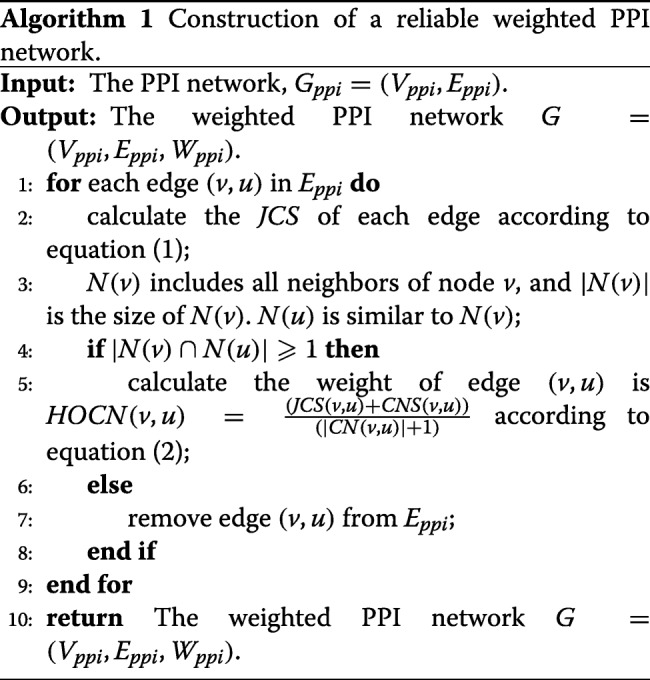


### Preliminary complex core identification

According to the latest research [31, 36, 47–50], a protein complex consists of core and periphery (also called attachment) proteins. A complex core is a small group of proteins that show high co-expression similarity and share high functional similarity, which is a key cellular role and the essential function for a protein complex [31, 35]. Unfortunately, due to the limitations of experimental methods, the functional information (gene ontology) of many proteins may be infeasible for the identification of protein complex cores [51]. However, the core proteins in the same complex core show a high level of functional similarity and have relatively more common neighbors among themselves than among other proteins in the PPI networks [15, 36, 51]. The biological functions of proteins are determined by their neighbors from the view of topological characteristics. This strategy is a good alternative in the absence of functional information. Thus, two proteins are assigned to the same protein complex core if they share many common neighbors. Because two proteins share many interaction neighbors, they are likely to carry out similar biological functions and be in the same complex core. Moreover, structural similarity could further assess the functional similarity between two proteins based on common neighbors and neighbourhood size [36, 47, 51].

As mentioned in “Preliminary” section, given a vertex *v*∈*V*_*ppi*_,*N*(*v*) stands for the set of all direct neighbors. Thus, the structural neighborhood of *v* is defined by Eq. (4):

4 $$ SN(v)=\{v\}\cup N(v),  $$

where *SN*(*v*) contains the node *v* and its immediate neighbors.

In the PPI networks, if two proteins have common neighbors, they may be functionally related. Furthermore, the structural similarity is used for normalizing common neighbors between two vertices in information retrieval [47]. This measure could be indirect functional similarity [36, 45]. As a result, structural similarity *SS* can be calculated by using the number of common neighbors which are normalized by the geometric mean of the neighbourhood size of vertex *v* and *w*. Therefore, the structural similarity *SS* between two neighbor proteins *v* and *u* is defined by Eq. (5):

5 $$ SS(v,w)=\frac{|SN(v)\cap SN(w)|}{\sqrt{|SN(v)|\cdot|SN(w)|}},  $$

when a vertex has a similar structure as that of one of its neighbors, their structural similarity is large. In additional, structural similarity is symmetric, i.e., *SS*(*v, w*)=*SS*(*w, v*). Obviously, the value of structural similarity is between (0,1]. Additionally, although the PPI networks have noise which will affect the clustering results, this scheme is not sensitive.

Based on these statements, we mine a subgraph in the neighborhood graph *G*_*v*_ based on structural similarity, which is used as a preliminary complex core and is written as *Core*(*PC*_*v*_). *Core*(*PC*_*v*_) consists of seed vertex *v* as the center and neighbors that should have high significance structural similarity with seed *v*. In addition, some biological experiments analyses, such as three-dimensional structure and yeast two-hybrid, have showed that the core proteins (vertices) in the same complex core are likely to be in direct physical contact with each other [31, 52]. Therefore, for each neighbor *u*∈*N*(*v*), if the value of structural similarity between it and seed *v* is larger than a prefixed threshold (e.g., 0.4), we select protein *u* as a core protein. The detail of this prefixed threshold selection will be introduced in Parameter selection section. The *Core*(*PC*_*v*_) of an identified complex *PC*_*v*_ is defined as the subgraph which is made of all the core proteins and their corresponding edges.

According to some relevant analysis results [15, 16, 31, 35, 36, 51], we try to summarize some possible conditions.

1. If the subgraph is small dense and reliable, its core proteins within the same protein complex core have relatively more interactions among themselves.
2. The core proteins in the same complex core are likely to be directly physical contact with each other.
3. The core proteins in the same complex core should have relatively more common neighbors than other non-core proteins.

According to these possible conditions and our studies, we take account of a preliminary complex core, named *Core*(*PC*_*v*_). It should satisfy the following three conditions.

1. The size of the preliminary complex core is larger than 2 and consists of core proteins, where all its core proteins directly connect with each other.
2. The core proteins of a complex core should have more reliable and heavier weights among themselves.
3. A complex core should have higher functional similarity.
4. The core proteins of a protein complex core could be shared with multiple protein complexes.

More specifically, we consider that each vertex *v*∈*V*_*ppi*_ is a seed to mine protein complex cores, and we compute *SS*(*v, w*) between *v* and each adjacent vertex *w*, when *SS*(*v, w*) is larger than or equal to a user-defined threshold (*ss*); then we take *w* as a core vertex to the preliminary complex core *Core*(*PC*_*v*_). Moreover, vertex *w* should be included into *Core*(*PC*_*v*_), because they are connected and share a similar structure. Each preliminary complex core *Core*(*PC*_*v*_) consists of seed vertex *v* and core vertices, and the value of *SS*(*v, w*) between seed vertex *v* and its direct neighbors is larger than or equal to a previously set threshold *ss*. Finally, we discard some redundancy preliminary complex cores and only retain preliminary complex cores whose size is greater than or equal to 2. The pseudo-code of this phase is shown in algorithm 2.


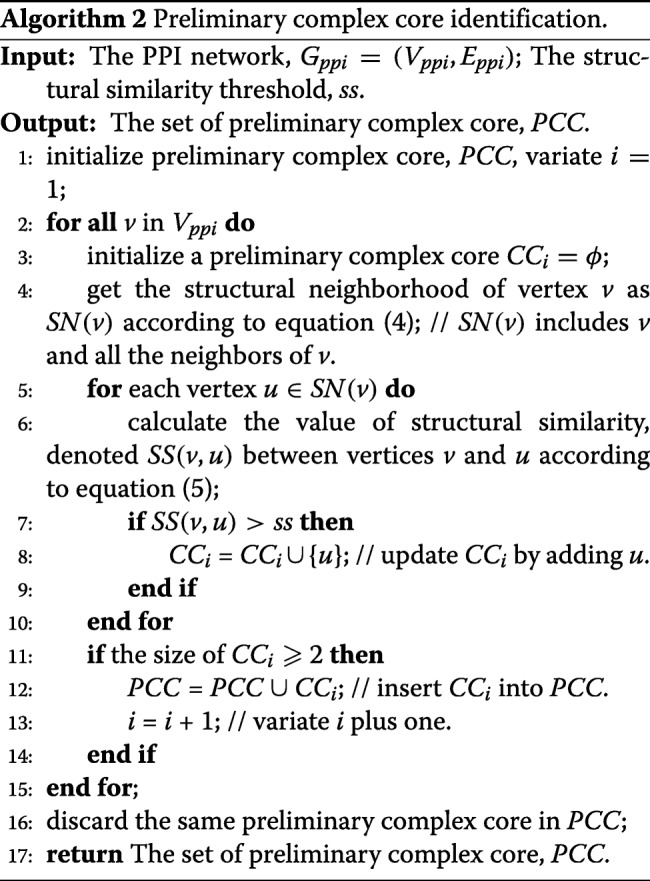


### Attachment protein detection

EWCA is used to detect the protein complex cores in the previous section. Next, we should identify the attachment proteins for each complex core to form the protein complex. The research of Gavin et al. [31] shows that attachment proteins are closely associated with core proteins within protein complexes and that a great degree of heterogeneity in expression levels and attachment proteins might represent nonstoichiometric components [31]. Also, attachment proteins are shared by two or more complexes and some overlapping proteins may participate in multiple complexes [53, 54]. According to Gavin et al.’s research [31] and our previous CALM algorithm [14], we know that a protein complex consists of a protein complex core and attachment proteins. Additionally, attachment proteins have two parts. One is peripheral proteins and the other is overlapping proteins. If the readers want to understand these concepts, please refer to ref [14, 31].

Based on the concepts of attachment proteins, attachment proteins contain could be grouped into two categories. The first category is peripheral proteins, and its main feature is that they only belong to one protein complex. In other words, they closely connect to the protein complex and belong to the most favored protein complexes. The second category is overlapping proteins, which, in contrast, belong to multiple protein complexes. According to our previous CALM algorithm statistics, the number of overlapping proteins in the known protein complexes [14] shows that a large fraction of proteins (called overlapping proteins) participate in multiple protein complexes. Here, we summarize the features of overlapping proteins. Overlapping proteins are proteins that belong to several protein complexes at the same time. Overlapping proteins connect to each protein complex with a different connection strength. We believe that dense protein-protein interaction in a protein complex is a key feature of protein complexes. Therefore, we adopt the average weighted degree of protein complexes which is based on the concept of density, to judge whether a protein is an overlapping protein or not.

Next, let us assume an identified complex, written as *PC*_*v*_. Here, we use a given a preliminary complex core *Core*(*PC*_*v*_)=(*V*_*core*_,*E*_*core*_) and a candidate attachment subset *CAP* to construct the identified complex *PC*_*v*_. We need to complete two tasks: one is to set up a subset *CAP*⊆*V*_*ppi*_ in which each protein *p*∈*CAP* is a candidate attachment protein for the identified protein complex *PC*_*v*_ and the other one is to decide which category each protein in *CAP* belongs to.

At first, for attachment proteins, we give two basic conditions: (1) attachment proteins should directly interact with the corresponding complex cores. (2) attachment proteins should connect with at least two or more core proteins with its complex core. If a protein *p* satisfies these conditions, it is selected as a candidate attachment protein, where protein *p* belongs to the neighbourhood of the preliminary complex core *Core*(*PC*_*v*_) and $N(p)\cap V_{core} \geqslant 2$. As a result, we have constructed a candidate attachment subset *CAP*. Next, we will discuss how to specifically identify the two categories. First of all, we consider a protein belong to that an overlapping protein should satisfy the following:

1. Overlapping proteins interact directly and closely with the corresponding complex cores.
2. The weighted out-connectivity of the complex core of the overlapping protein is greater than the weighted in-connectivity of the complex core.
3. Overlapping proteins weakly interact with the corresponding complex core relative to the internal interactions within the corresponding complex core.
4. Overlapping proteins are not unique to a protein complex; instead, they may be present in more than one complex.

According to these conditions, we let a candidate attachment protein *p* of an identified complex *PC*_*v*_ be an overlapping protein in a candidate attachment set *CAP*, that is, *p*∈*Overlapping*(*PC*_*v*_):

1. The weighted out-connectivity of *p* with respect to *Core*(*PC*_*v*_) is greater than or equal to the weighted in-interactions of *p* with respect to *Core*(*PC*_*v*_), given by: $weight_{out}(p,Core(PC_{v}))\geqslant weight_{in}(p,Core(PC_{v}))$.
2. The weighted in-interactions of *p* with respect to *Core*(*PC*_*v*_) is at least half of the average weighted in-interactions of all core vertices in *Core*(*PC*_*v*_), given by: $d_{weight}(p,Core(PC_{v}))\geqslant \frac {1}{2} weight_{avg}(Core(PC_{v}))$.

Here, *d*_*weight*_(*p, Core*(*PC*_*v*_)) is the total weight interactions of *p* with core proteins in *Core*(*PC*_*v*_), given by $d_{weight}(p,Core(PC_{v}))=\sum _{p\notin V_{core},t\in V_{core}} weight(p,t)$. *weight*_*avg*_(*Core*(*PC*_*v*_)) is the average of the weighted interactions of all core proteins within the complex core *Core*(*PC*_*v*_), calculated by $weight_{avg}(Core(PC_{v}))=\frac {2*\sum _{(v,u)\in E_{core}} weight(v,u)}{|V_{core}|}$, where |*V*_*core*_| is the number of proteins in the *Core*(*PC*_*v*_) and $\sum _{(v,u)\in E_{core}} weight(v,u)$ represents the total weight of interactions in the protein complex core *Core*(*PC*_*v*_). If a protein satisfies these conditions, we suppose that it belongs to protein complex *PC*_*v*_ at the same time and make it an overlapping protein.

Second, when we have obtained all overlapping proteins from candidate attachment set *CAP*, we next obtain a candidate peripheral protein subset, *CP*(*PC*_*v*_), which is a difference set, given by *CAP*−*Overlapping*(*PC*_*v*_). We consider that a peripheral protein should satisfy the following:

1. Peripheral proteins are not overlapping proteins.
2. The weighted in-connectivity of the complex core of the peripheral proteins is greater than the weighted out-connectivity of the complex core.
3. Peripheral proteins closely interact with corresponding complex core relative to the interaction of other non-member proteins with the corresponding complex core.
4. Peripheral proteins only belong to a protein complex.

Considering these criteria, we let a candidate attachment protein *p* of an identified complex *PC*_*v*_ be a peripheral protein in a candidate peripheral protein subset *CP*(*PC*_*v*_), that is, *p*∈*Periphery*(*PC*_*v*_):

1. The weighted in-interactions of *p* with respect to *Core*(*PC*_*v*_) is greater than the weighted out-connectivity of *p* with respect to *Core*(*PC*_*v*_) and is written by: *weight*_*in*_(*p, Core*(*PC*_*v*_))>*weight*_*out*_(*p, Core*(*PC*_*v*_)).
2. The weighted in-interactions of *p* with respect to *Core*(*PC*_*v*_) is greater than the average weight of interactions of all all candidate peripheral proteins with *Core*(*PC*_*v*_) and is given by: $weight_{in}(p,Core(PC_{v}))\geqslant weight_{avg}(CP(PC_{v}))$.

Here, $weight_{avg}(CP(PC_{v}))=\frac {\sum _{c\in CP(PC_{v})}weight_{in}(c,Core(PC_{v}))}{|CP(PC_{v})|}$ is the average weight of interactions of the entire candidate peripheral protein subset *CP*(*PC*_*v*_) with *Core*(*PC*_*v*_).

Combining the peripheral proteins and overlapping proteins, we form the final set of attachment proteins of protein complex core *Core*(*PC*_*v*_), that is:

6 $$ {{} \begin{aligned} Attachment(PC_{v})\,=\,\! \{Periphery(PC_{v})\!\cup\! Overlapping(PC_{v})\!\}. \end{aligned}}  $$

The more detailed pseudo-codes of this phase is shown in Algorithm 3.


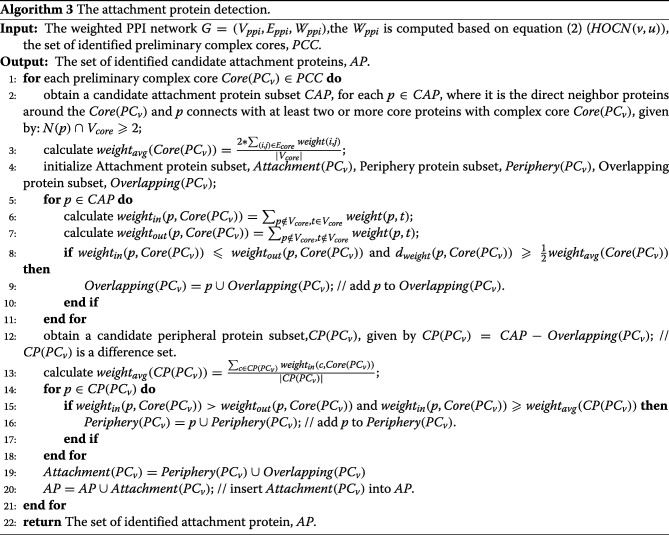


### Protein complex formation

After we have obtained the set of identified preliminary complex cores and the set of identified candidate attachment protein, we combine a preliminary complex core and its attachment proteins and form the final set of unique complex (*PC*_*v*_), i.e.,

7 $$ PC_{v}= \{Core(PC_{v})\cup Attachment(PC_{v})\},  $$

Furthermore, we discard protein complexes with a size of less than 3 proteins. Moreover, because different protein complex cores may produce the same identified protein complexes, some redundant protein complexes are identified. Thus, some protein complexes are completely overlap with each other, which means that only one of them is retained while the others are removed as redundant protein complexes, The detailed pseudo-code of this phase is shown in Algorithm 4.


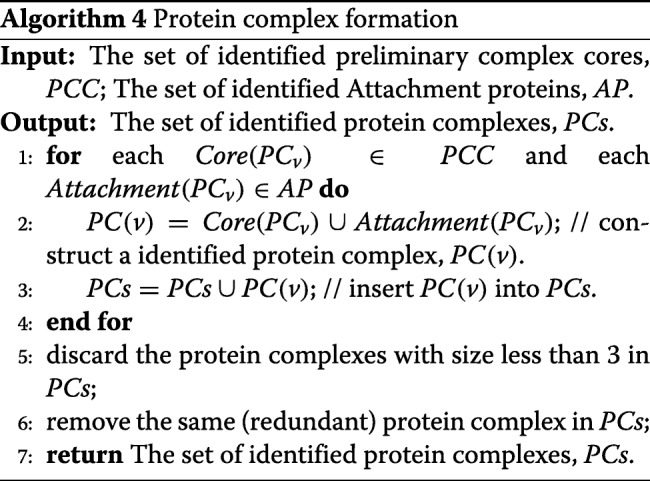


## Datasets and evaluation metrics

### Experimental datasets

We do the experiment on the three PPI networks of *S*.*cerevisiae* extracted from the PPI Networks DIP [55], BioGRID [56] and Yeast [57], respectively. The general properties of the datasets are shown in Table 1. For human, the PPI network is constructed by combining the data from Human [57]. For more detail about Yeast and Human datasets, see the Ref [57].

**Table 1:** The details of PPI networks used in experiments

Dataset	Number of node	Number of edge	Density
DIP	4930	17202	0.00141572191
BioGRID	5640	59748	0.00000315987
Yeast	6194	74826	0.00390130805
Human	15459	144687	0.00121094608

For yeast, three reference sets of protein complexes are used in our experiments. One set comprises of hand-curated complexes from CYC2008 [23] and the other set is NewMIPS which generated by MIPS [58], Aloy [59] and the Gene Ontology (GO) annotations in the SGD database [60]. The last *Yeast complexes* [57] come from the Wodak database (CYC2008) [23], PINdb and GO complexes. For human, *Human complexes* [57] are collected from the Comprehensive Resource of Mammalian protein complexes (CORUM) [61], protein complexes are annotated by GO [62], Proteins Interacting in the Nucleus database (PINdb) [63] and KEGG modules [64]. For all of them, we only keep the complexes with size no less than 3. The general properties of the reference complex sets are shown in Table 2.

**Table 2:** General properties of the standard protein complexes

Datasets	Number of protein complexes	Protein coverage	Avg size
CYC2008	236	1628	4.71
NewMIPS	328	1171	14.93
Human complexes	2289	6206	8.57
Yeast complexes	1045	2773	8.92

### Evaluation metrics

There are several evaluation metrics that can be used to perform comprehensive comparisons, such as recall, precision, F-measure and so on. Here, we employ them as previously suggested by study [13, 16, 65]. Overall, there are five types of evaluation metrics used to evaluate the quality of the identified complexes and compare the overall performance of the identification methods. The definitions of these evaluation measures are introduced as follows.

#### Recall, precision and F-measure

Generally speaking, clustering results are evaluated in terms of recall, precision, and F-measure. Recall [66] is termed the true positive rate or sensitivity, and it is the ratio of the number of proteins in both identification complexes and reference complexes to the number of proteins in the reference complexes. Precision [66] is the ratio of the maximal number of common vertices in both identified complexes and reference complexes to the number of vertices in identified complexes. Meanwhile, F-measure is a harmonic measure according to recall and precision [66] and it is used for evaluating the accuracy of the identified complexes. The F-measure could evaluate not only the accuracy of identified complexes matching reference complexes but also the accuracy of protein complexes matching identified complexes.

The identified complexes *P*={*p*_1_,*p*_2_,...,*p*_*k*_} is generated by identified method, and *R*={*r*_1_,*r*_2_,...,*r*_*l*_} is the reference complexes for any identified complex *p*_*i*_ and reference complex *r*_*j*_. First, we introduce the neighborhood affinity (*NA*(*p*_*i*_,*r*_*j*_)) between the identified protein complexes and reference complexes, which is presented as follows [16, 65, 67]:

8 $$ \begin{aligned} NA(p_{i},s_{j})=\frac{|N_{p_{i}}\cap N_{s_{j}}|^{2}}{|N_{p_{i}}| \times |N_{s_{j}}|}, \end{aligned}  $$

Here, the neighborhood affinity *NA*(*p*_*i*_,*r*_*j*_) is defined to measure the similarity between identified complexes and reference complexes, and it quantizes the closeness between them. $|N_{p_{i}}|$ is the size of the identified complex, $|N_{r_{j}}|$ is the size of the reference complex, and $|N_{p_{i}}\cap N_{r_{j}}|$ is the number of common proteins from the identified and reference complexes. The larger the value of *NA*(*p*_*i*_,*r*_*j*_) is, the more possible two complexes closer are. If *NA*(*p*_*i*_,*r*_*j*_)≥*t*, then the *p*_*i*_ is considered to be matched with *r*_*j*_, where *t* is a predefined threshold. In this paper, we also set t = 0.2, which is consistent with previous studies [16, 65].

After the neighborhood affinity *NA*(*p*_*i*_,*r*_*j*_) has been defined, we will give the definition of recall, precision and F-measure. We assume that *P* and *R* are the set of identified complexes and real reference complexes, respectively. *N*_*mr*_ is the number of reference complexes that match at least an identified complex, i.e. *N*_*mr*_=|{*r*|*r*∈*R*,∃*p*∈*P, NA*(*r, p*)≥*t*}|. *N*_*mp*_ is the number of correct identification complexes that match at least a real protein complex, i.e., *N*_*mp*_=|{*p*|*p*∈*P*,∃*r*∈*R, NA*(*p, r*)≥*t*}|. Recall and precision are defined as follows [68]:

9 $$ \begin{aligned} Recall = \frac{N_{mr}}{|R|}, \end{aligned}  $$

and

10 $$ \begin{aligned} Precision = \frac{N_{mp}}{|P|}. \end{aligned}  $$

In general, a larger protein complex has the higher recall, while a smaller protein complex has higher precision. Therefore, the F-measure is defined as the harmonic mean of recall and precision, which The corresponding formulas are shown as follows [69]:

11 $$ \begin{aligned} F-measure=\frac{2\times(Precision\times Recall)}{Precision + Recall} \end{aligned}  $$

#### Coverage rate and mMR

The coverage rate is use for assessing how many proteins in the reference complexes could be covered by the identified complexes [70, 71]. In detail, when the set of reference complexes *R* and the set of identified complexes *P*, are given the |*R*|×|*P*| matrix *T* is constructed, where each element *max*{*T*_*ij*_} is the largest number of proteins in common between the *i*th reference complex and the *j*th identified complex. The coverage rate is defined as:

12 $$ \begin{aligned} CR=\frac{\sum_{i=1}^{|R|}max\{T_{ij}\}}{\sum_{i=1}^{|R|}N_{i}}, \end{aligned}  $$

where *N*_*i*_ is the number of proteins in the *i*th standard complex.

The MMR metric, which is strongly recommended by Nepusz et al. [13], measures the number of maximal matching between reference complexes and identified protein complexes. As discussed by the authors, it penalizes the methods that tend to split a reference complex into more than one part in the identified complexes. To do so, a bipartite graph is composed by two sets of vertices, and the edge between an identified complex and a reference complex is weighted by the matching score of *NA*(*A, B*) (see Eq. (8)). The MMR score between the identified complex and the reference complex is the total weight of edges, selected by the maximum weighted bipartite matching and divided by the number of known complexes. For more details about computing MMR, please refer to references [13].

The above three kinds of metrics are independent and can work together to evaluate the performance of protein complex identification methods [13].

#### Analysis of function enrichment

Moreover, because of laboratory-based experiments limitation, the known protein complexes are incomplete. Therefore, many researchers [7, 72] annotate their main biological functions by using *p*-value formulated as Eq. (13). We also adopt function enrichment test to demonstrate the biological significance of the identified protein complexes. Given an identified protein complex containing *C* proteins, *p*-value is used for calculating the probability of observing *m* or more proteins from the complex by chance in a biological function shared by *F* proteins from a total genome size of *N* proteins:

13 $$ p-value=1-\sum_{i=0}^{m-1} \frac{{{F}\choose{i}}{{N-F}\choose{C-i}}}{{{N}\choose{C}}}.  $$

Here, where *N* is the total number of vertices in the PPI networks, *C* is the size of the identified complex, *F* is the size of a functional group, and *m* is the number of proteins of the functional group in the identified complex. The *p*-value is calculated on biological process ontologies. The smaller the *p*-value of a protein complex is, the more biological significance of the protein complex is. In general, if the *p*-value is lower than 0.01, the protein complex is considered to be significant.

## Results

### Comparison between different methods

To demonstate the effectiveness of EWCA in identifying protein complexes, we compare EWCA with twelve existing state-of-the-art protein complex identification algorithms including MCL, CFinder, Core, DPClus, COACH, SPICi, ClusterONE, PEWCC, GMFTP, CMC, ProRank+ and DPC. To be fair for each compared method, we follow the strategy used in [6, 13], the optimal parameters of the reference complexes are set to generate the best result for each compared method, and the optimal parameters with respect to the reference complexes are set to generate its best result or follow as suggested by the authors. More details and the selection of parameters for all the compared methods are supplied in website (https://github.com/RongquanWang/EWCA/SupplementaryMaterial.docx). Here we chose these parameters that can maximize the value of F-measure, because it could fully balance the performance of all methods. Moreover, the comparison results between EWCA and other methods are shown in Tables 3 and 4, which is the overall performance of each methods based on recall, precision, F-measure, MMR and CR.

**Table 3:** Performance comparison with other methods based on NewMIPS

Algorithms	Recall	Precision	F-measure	MMR	CR
BioGRID					
MCL	0.2896	0.2011	0.2374	0.0726	0.2995
CFinder	0.5914	0.1960	0.2944	0.2801 ^3*rd*^	0.4402
Core	0.5609	0.1488	0.2352	0.1437	0.5882
DPClus	0.6951	0.1741	0.2785	0.201	0.5597
CMC	0.8109 ^1*st*^	0.2731	0.4086	0.3175 ^2*nd*^	0.4954
COACH	0.7256	0.2581	0.3807	0.2525	0.6322 ^3*rd*^
SPICi	0.4969	0.3725	0.4258	0.1304	0.4378
ClusterONE	0.5914	0.3130	0.4093	0.1917	0.5311
PEWCC	0.4512	0.5943 ^2*nd*^	0.5129 ^3*rd*^	0.1889	0.4119
ProRank+	0.4817	0.7131 ^1*st*^	0.5750 ^2*nd*^	0.241	0.4763
GMFTP	0.7530 ^3*rd*^	0.2830	0.4114	0.2551	0.5186
DPC	0.6310	0.3050	0.4112	0.2312	0.6332 ^2*nd*^
EWCA	0.7561 ^2*nd*^	0.5821 ^3*rd*^	**0.6578** ^1 *st*^	**0.3764** ^1 *st*^	**0.6497** ^1 ***st***^
DIP					
MCL	0.4908	0.1783	0.2616	0.1255	0.3271
CFinder	0.5762	0.2408	0.3396	0.2128	0.2403
Core	0.4420	0.1746	0.2504	0.1249	0.3902
DPClus	0.6067 ^3*rd*^	0.1392	0.2265	0.1626	0.3356
CMC	0.5932	0.4152	0.4885	0.2499 ^2*nd*^	**0.5736** ^1 ***st***^
COACH	0.5731	0.5106 ^2*nd*^	0.5401 ^2*nd*^	0.2006	0.3351
SPICi	0.4847	0.2473	0.3275	0.1095	0.3191
ClusterONE	0.4054	0.3020	0.3462	0.1178	0.2417
PEWCC	0.5670	0.4822	0.5212 ^3*rd*^	0.2297 ^3*rd*^	0.3280
ProRank+	0.4085	**0.6657** ^1 *st*^	0.5063	0.1669	0.2444
GMFTP	0.6981 ^2*nd*^	0.2755	0.3951	0.2228	0.4043 ^2*nd*^
DPC	0.4908	0.4389	0.4634	0.1717	0.3305
EWCA	**0.7012** ^1 *st*^	0.4990 ^3*rd*^	**0.5830** ^1 *st*^	**0.3094** ^1 ***st***^	0.3982 ^3*rd*^

NOTE: The highest value in each column is shown in bold

**Table 4:** Performance comparison with other methods based on CYC2008

Algorithms	Recall	Precision	F-measure	MMR	CR
BioGRID					
MCL	0.3516	0.2268	0.2758	0.1245	0.5310
CFinder	0.5720	0.1637	0.2546	0.3115	0.6135
Core	0.5847	0.1527	0.2422	0.2081	0.8058
DPClus	0.7839	0.1978	0.3158	0.304	0.8160
CMC	**0.8644** ^1 *st*^	0.2677	0.4088	**0.4375** ^1 *st*^	0.7639
COACH	0.7669	0.2488	0.3757	0.3042	**0.8750** ^1 ***st***^
SPICi	0.5127	0.4039	0.4518	0.1997	0.6065
ClusterONE	0.6610	0.3487	0.4565	0.2734	0.7569
PEWCC	0.4025	0.5374 ^3*rd*^	0.4603 ^3*rd*^	0.2142	0.5431
ProRank+	0.4153	**0.6622** ^1 *st*^	0.5104 ^2*nd*^	0.246	0.5850
GMFTP	0.7838 ^3*rd*^	0.2914	0.4249	0.3913 ^3*rd*^	0.7956
DPC	0.7033	0.2874	0.4081	0.2643	0.8616 ^3*rd*^
EWCA	0.8093 ^2*nd*^	0.5793 ^2*nd*^	**0.6752** ^1 *st*^	0.4351 ^2*nd*^	0.8718 ^2*nd*^
DIP					
MCL	0.5169	0.1847	0.2721	0.1899	0.4892
CFinder	0.5508	0.2398	0.3342	0.2788	0.3807
Core	0.4618	0.1818	0.2609	0.2033	0.5317
DPClus	0.6651 ^3*rd*^	0.1518	0.2473	0.2610	0.5184
CMC	0.5932	0.4125	0.4866	0.2501	0.5755 ^3*rd*^
COACH	0.5423	0.5167 ^3*rd*^	0.5292 ^2*nd*^	0.2764	0.4879
SPICi	0.5000	0.2769	0.3564	0.1665	0.4600
ClusterONE	0.4279	0.3343	0.3753	0.1840	0.3750
PEWCC	0.5296	0.4852	0.5064 ^3*rd*^	0.2847 ^3*rd*^	0.4682
ProRank+	0.3771	**0.6923** ^1 *st*^	0.4883	0.2029	0.3293
GMFTP	0.6652 ^2*nd*^	0.2664	0.3804	0.3315 ^2*nd*^	**0.6085** ^1 ***st***^
DPC	0.4872	0.4598	0.4731	0.2146	0.4828
EWCA	**0.7076** ^1 *st*^	0.5239 ^2*nd*^	**0.6020** ^1 *st*^	**0.3766** ^1 ***st***^	0.5806 ^2*nd*^

NOTE: The highest value in each column is shown in bold

What’s more, EWCA achieves almost the highest F-measure and MMR is also the highest through four combinations of the two PPI datasets and the two reference complexes. Please note that we have removed identified complexes with having two or less proteins, and we do not any supply biological data (e.g., Go annotations) in EWCA method and other compared methods. The bold values is the best result in comparison with other methods. In fact, F-measure is the harmonic mean of recall and precision. Obviously, the higher F-measure is better.

Table 3 shows the comprehensive comparison results on the unweighted networks in terms of five criterion by using the NewMIPS complexes. EWCA achieves the highest F-measure and MMR, which are compared with the other methods across all two combinations of the two PPI datasets. It is obvious that EWCA could identify protein complexes more accurate. In Table 3, when using BioGRID dataset as input PPI network and NewMIPS as reference complexes, EWCA obtains the highest F-measure that is 0.6578, that is higher better balance between recall and precision. Similar, EWCA is the highest value in terms of MMR and CR. As shown in Table 3, EWCA achieves the highest recall of 0.7012, F-measure of 0.5830 and MMR of 0.3094 in the DIP PPI network, which obviously outperforms other methods. Meanwhile, EWCA obtains a higher MMR than other methods, and it indicates that the identification of protein complexes by EWCA can obtain a better maximal one-to-one mapping to NewMIPS complexes. In short, Table 3 shows that EWCA obviously outperforms other methods on the NewMIPS complexes.

Table 4 shows the overall comparative results on the unweighted networks using the CYC2008 complexes. In Table 4, when the PPI dataset is BioGRID, EWCA achieves the highest F-measure of 0.6752, however the second highest ProRank+ is just 0.5104. It is the main difference between EWCA and other methods, which means EWCA has the absolutely advantage. Compared with other methods, EWCA’s other criterion is just a little lower than the highest of other methods. Secondly, when we compare EWCA with the other methods by using DIP PPI network. Similarly, EWCA still outperforms others methods as shown in Table 4. The experimental results show that EWCA achieves both the highest recall of 0.7076, the highest F-measure of 0.6020 and the highest MMR of 0.3766 in the DIP PPI network. Meanwhile, it indicates that our identified protein complexes could match to reference complexes, which is significantly superior to the other methods. Furthermore, compared with CR, EWCA is a little lower than the best GMFTP on DIP PPI network. Furthermore, for other assessment measure, EWCA is very close the best in DIP dataset as shown in Table 4. Meanwhile, the experimental results by using the CYC2008 as reference complexes are basically consistent with using the NewMIPS as reference complexes.

In summary, EWCA achieves the better performance on two PPI network, which is competitive or superior to the existing protein complexes identification methods. Especially, EWCA achieves a consistently better F-measure and MMR than the other twelve methods. Tables 3 and 4 present the comparison results under two reference complexes.

### Analysis of function enrichment

Since the reference complexes are incomplete, to further validate the effectiveness of EWCA method, we investigate the biological significance of our identified protein complexes. Each identified complex is associated with a *p*-value (as formulated in Eq. (13)) for gene ontology (GO) annotation. In general, an identified complex by different identification methods is considered biologically significant if its *p*-value is less than 1E-2. Meanwhile, an identified complex has a lower *p*-value, the more statistically biological significance. We calculate the *p*-value of identified complexes based on biological process ontologies by using the web service of GO Term Finder (https://www.yeastgenome.org/goTermFinder) [73] which is provided by SGD [74]. Here, for each identification complex, we use the smallest *p*-value over all possible gene ontology term to represent its functional homogeneity. Besides analyzing the protein complexes identified by EWCA, we also calculate the *p*-value of protein complexes identified by CMC, PEWCC, GMFTP, COACH, ProRank+ and DPC whose size are greater than or equal to 3, respectively. Selecting the above methods to compare with EWCA is because all of them obtained better performances in two test PPI networks as shown in Tables 3 and 4.

The results of *p*-value test for CMC, PEWCC, GMFTP, COACH, ProRank+, DPC and EWCA are presented in Table 5. To compare the biological significance of different algorithms, the number of identified complexes, the number of identified complexes and the proportion of identified complexes by various methods whose *p*-value falls within different value ranges are calculated for each algorithm. Most of previous algorithms only take account of the proportion of identified complexes. However, the *p*-value of protein complexes identified has close relationship with their size [16]. Therefore, we should consider both the number of identified complexes and the proportion of identified complexes to analyze function enrichment of identified protein complexes. As the Table 5 shows, on the BioGRID dataset, the proportion of significant protein complexes identified by EWCA is 96.62 percent, which is about 1 percentage point lower than the best method COACH and 0.97 percentage point lower than the second best method ProRank+. It may be due to the fact that EWCA detects many more protein complexes than COACH and ProRank+ and the size of identified protein complexes by EWCA is relatively smaller than other algorithms, such as ProRank+. However, it is obvious that the number of identified protein complexes by EWCA is 1341, which is maximum and it is far more than COACH and ProRank+.

**Table 5:** Function enrichment analysis of protein complexes detected from different datasets

Dataset	Algorithms	PC	<E-15	[E-15,E-10)	[E-10,E-5)	[E-5,0.01)	Significant
BioGRID	CMC	1113	125(11.23%)	89(7.99%)	258(23.18%)	360(32.34%)	832(74.76%)
	PEWCC	387	181(46.77%)	64(16.53%)	83(21.44%)	46(11.88%)	374(96.65%)
	GMFTP	597	73(12.22%)	59(9.88%)	156(26.13%)	161(26.96%)	449(75.21%)
	COACH	166	76(45.78%)	32(19.27%)	38(22.89%)	16(9.63%)	162(97.60%)
	ProRank+	746	479(64.20%)	105(14.07%)	97(13.00%)	47(6.30%)	18(97.59%)
	DPC	2167	596(27.50%)	166(7.66%)	290(13.38%)	569(26.25%)	1621(74.81%)
	EWCA	1388	658(47.40%)	211(15.20%)	299(21.54%)	173(12.46%)	1341(96.62%)
DIP	CMC	303	1(0.33%)	8(2.64%)	58(19.14%)	77(25.41%)	144(47.53%)
	PEWCC	676	78(11.53%)	117(17.30%)	278(41.12%)	132(19.52%)	605(89.50%)
	GMFTP	548	43(7.84%)	36(6.56%)	105(19.16%)	166(30.29%)	350(63.69%)
	COACH	329	21(6.38%)	25(7.59%)	66(20.06%)	32(9.72%)	144(43.68%)
	ProRank+	338	74(21.89%)	77(22.78%)	126(37.27%)	42(12.42%)	319(94.38%)
	DPC	622	72(11.57%)	113(18.16%)	197(31.67%)	176(28.29%)	558(89.72%)
	EWCA	964	188(19.50%)	126(13.07%)	319(33.09%)	236(24.48%)	870(90.15%)

NOTE: Table 5 lists the number percentage of protein complexes detected by CMC, PEWCC, GMFTP, COACH, ProRank+, DPC and EWCA in the PPI network whose *p*-value falls within different value ranges. In order to analyze functional enrichment, we should take into account of two values. For example, in the DIP dataset, in the fourth column of the fourteenth row 188 times 19.50% is 36.66 which is the highest value in this column that means EWCA is the best among these methods. Here, from the fourth column to the seventh column the larger value is, the better functional enrichment is

On the DIP dataset, the proportion of significant protein protein complexes identified by EWCA is 90.15 percent, which is about 4 percentage point lower than the best method ProRank+. Meanwhile, the number of identified protein complexes by EWCA is also maximum. Similarly, the number of identified protein complexes by CMC and GMFTP in BioGRID dataset is 1113, 2167, respectively. The number of identified protein complexes by PEWCC and DPC in BioGRID dataset is 676 and 622, respectively. Generally, the smaller the number of identified protein complexes is, the higher the proportion of significant complexes is. In fact, the number of identified protein complexes by CMC, GMFTP and PEWCC is much smaller than EWCA. However, they have the percentage of significant protein complexes is relatively lower than EWCA method. All in all, EWCA has more practical and biological significant than other methods in terms of the number of identified protein complexes and the proportion of identified complexes. According to their *p*-value, those identified protein complexes by EWCA has a higher possibility to be identified as real protein complexes through laboratory experiments in the future.

To further reveal the biological significance of identified complexes, five identified protein complexes with very low *p*-values provide by EWCA method with different datasets are presented in Table 6, which lists the *p*-values (Biological Process) of protein complexes, Cluster frequency and Gene Ontology term. The third column of Table 6 shows the cluster frequency. From this column, we can see that many of our identification protein complexes match well with the Gene ontology term. The *p*-value of identified complexes in Table 6 is very low, which further demonstrates that the protein complexes identified have high statistical significance.

**Table 6:** Some example of identified complexes with low *p*-value detected by EWCA method on different datasets

Dataset	ID	*P*-value(BP)	Cluster frequency	Gene ontology term
BioGRID	1	8.83e-108	62 of 66 genes, 93.9%	mRNA splicing, via spliceosome
	2	2.68e-106	70 of 71 genes, 98.6%	cytoplasmic translation
	3	1.09e-80	78 of 92 genes, 84.8%	chromatin organization
	4	2.11e-72	55 of 88 genes, 62.5%	ribosomal large subunit biogenesis
	5	2.48e-78	83 of 102 genes, 81.4%	ribosome biogenesis
DIP	1	4.62e-32	14 of 16 genes, 87.5%	mRNA polyadenylation
	2	1.54e-31	24 of 25 genes, 96.0%	mRNA processing
	3	2.96e-25	15 of 23 genes, 65.2%	maturation of LSU-rRNA from tricistronic rRNA transcript
	4	1.80e-28	16 of 18 genes, 88.9%	histone acetylation
	5	5.58e-29	12 of 13 genes, 92.3%	ATP biosynthetic process

Furthermore, we discover many identified protein complexes with cluster frequency of 100%. Here, let’s take 5 examples with *p*-value less than E-19 are listed in Table 7. Such identified protein complexes are probably real protein complexes, which also provide meaningful references to the related researchers.

**Table 7:** Ten protein complexes with cluster frequency being 100% on different datasets

Datasets	ID	*P*-value(BP)	Cluster Frequency	Gene ontology term
BioGRID	1	1.76e-75	46 of 46 genes, 100.0%	RNA splicing
	2	1.42e-43	16 of 16 genes, 100.0%	tRNA transcription
	3	5.77e-40	23 of 23 genes, 100.0%	mRNA transport
	4	1.36e-32	14 of 14 genes, 100.0%	ergosterol biosynthetic process
	5	2.24e-30	20 of 20 genes, 100.0%	DNA replication
DIP	1	4.68e-26	10 of 10 genes, 100.0%	anaphase-promoting complex-dependent catabolic process
	2	1.06e-31	19 of 19 genes, 100.0%	mRNA splicing, via spliceosome
	3	7.37e-27	21 of 21 genes, 100.0%	mRNA metabolic process
	4	8.64e-24	15 of 15 genes, 100.0%	mitochondrial translation
	5	2.51e-19	10 of 10 genes, 100.0%	ncRNA transcription

## Discussion

### Parameter selection

In this experiment, we introduce an user-defined parameter structural similarity (*ss*) and study its effect to identifying protein complexes. For yeast, protein complexes are identified from the two yeast PPI datasets including DIP and BioGRID in Table 1. The performance is evaluated in terms of precision, recall, F-measure, MMR and CR, which are calculated by using NewMIPS and CYC2008 as reference complexes.

**Fig. 3:**
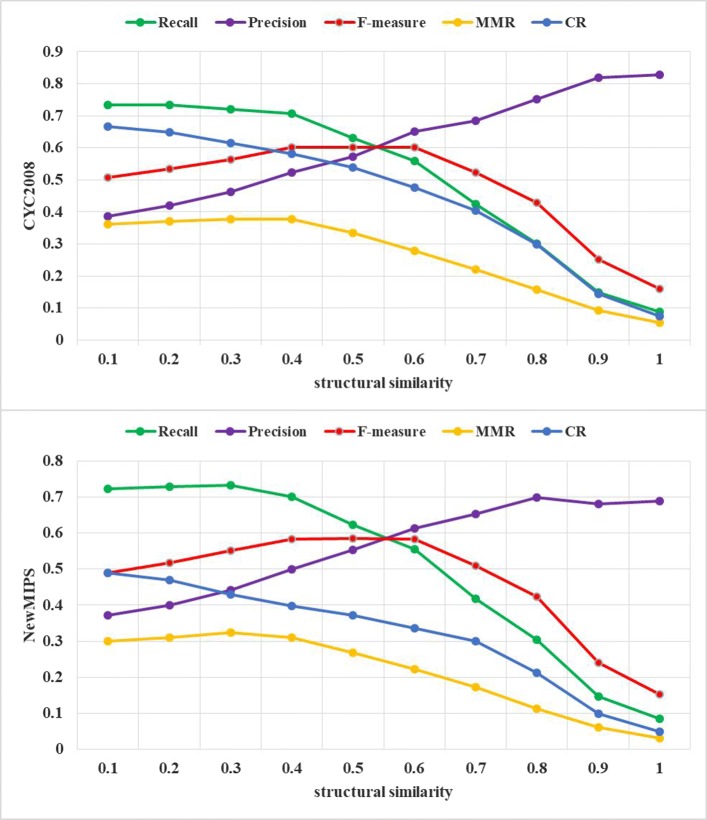
The effect of *ss*. Performance of EWCA on protein complex identification with different values of structural similarity threshold values of *ss* is measured by all evaluation meterics, with respect to CYC2008 and NewMIPS standard complex sets. The x-axis denotes the value of structural similarity and the y-axis denotes some evaluation metrics in DIP dataset. The F-measure is maximised at *ss*=0.4 for unweighted DIP dataset

**Fig. 4:**
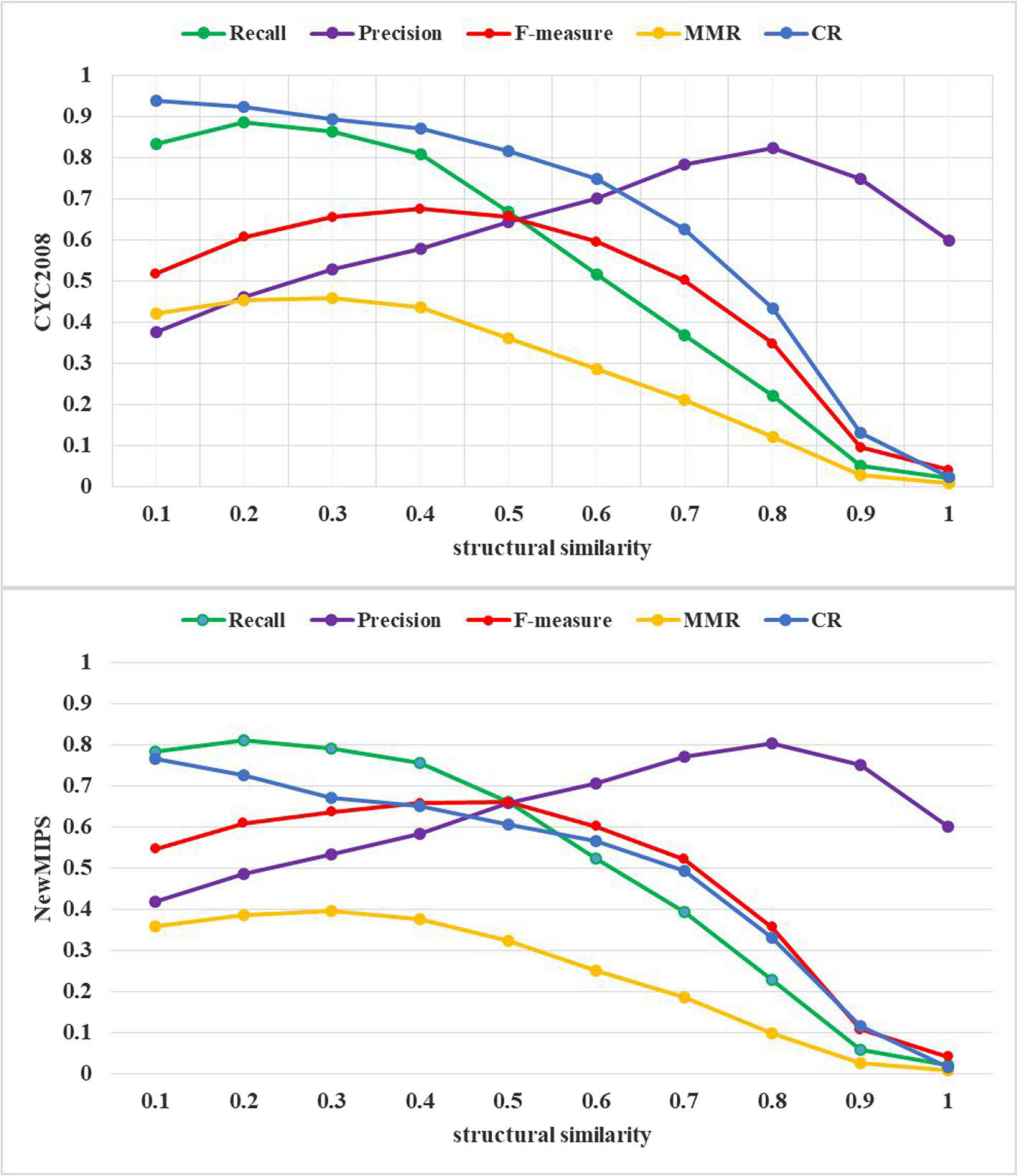
The effect of *ss*. Performance of EWCA with different structural similarity threshold *ss* is measured by all evaluation meterics, with respect to CYC2008 and NewMIPS standard complex sets. The x-axis denotes the value of structural similarity and the y-axis denotes evaluation metrics in BioGRID dataset. The F-measure is maximised at *ss*=0.4 on unweighted BioGRID dataset

To investigate the effect of the parameter *ss* on performance of EWCA, we evaluate the identification accuracy by setting different values of *ss* and we change the value of parameter *ss* from 0.1 to 1.0 with 0.1 increment. It is obvious that *ss* is allowed when *ss*> 0 and is not allowed when *ss* = 0. Figures 3 and 4 show the performance of EWCA method fluctuates under various *ss* and the results on DIP dataset and BioGRID dataset are shown separately. Figures 3 and 4 indicate that EWCA gets the better performance when *ss* is assigned 0.4.

As shown in Figs. 3 and 4, with the increase of *ss*, the value of recall, MMR and CR decrease but the value of precision increases. It is shown almost similar trends in all cases. Furthermore, we study the behaves of EWCA in terms of F-measure. Notably, in DIP dataset, the value of F-measure increases gradually with the increase of *ss* until *ss* = 0.4. Here, when CYC2008 and NewMIPS reference complexes are used, the maximum value of F-measure is 0.6020 and 0.5830, respectively. As the gradual increase of *ss*, the value of F-measure shows different change trends, which are all below *ss* = 0.4. For the DIP dataset, we set *ss* = 0.4. Similarly, in the BioGRID dataset, the value of F-measure increases as *ss* increasing and the value reach up to 0.6752 and 0.6578 by using CYC2008 and NewMIPS reference complexes when *ss* = 0.4, which is the optimal value as shown in Fig. 4. In the rest of experiment, we set *ss* = 0.4 for obtaining experimental results.

As a result, we recommend that the suitable range of *ss* would be from 0.4 to 0.6. Because the value of F-measure does not change significantly in this range.

### Time complex analysis

In this section, we analyze the computational complexity of EWCA algorithm. All experiments are run on an Intel(R) Core(TM) i7-4790 CPU @ 3.60GHz computer with 12.0 GB memory. For simplicity, we run all the programs with their default parameter. Meanwhile, all reported run times are clock times for running protein complexes identification methods. Furthermore, because the accuracy of protein complexes identification methods is most important. Therefore, we only select these comparison methods with having high accuracy according to Tables 3 and 4 to compare efficiently.

We present an analysis of the computation complexity of the algorithm EWCA. Given a graph with *m* edges and *n* vertices, EWCA first executes Algorithm 1. For each edge, EWCA computes the weight of the edge. For one vertex, EWCA visits its direct neighbors. Here, we use an adjacency list which is a data structure where each vertex has a list which includes all its neighbor vertices. The cost of neighborhood query is proportional to the number of neighbors, that is, the degree of query vertex. Therefore, the total cost is *O*(*deg*(*v*_1_)+*deg*(*v*_2_)+*deg*(*v*_*i*_)+...+*deg*(*v*_*n*_)), where *deg*(*v*_*i*_),*i*=1,2,*i*,...,*n* is the degree of vertex *v*_*i*_. If we sum all the vertex degrees in *G*, we count each edge exactly twice: *O*(2∗*m*). Meanwhile, each edge has two vertices. Thus the computation complexity of Algorithm 1 is *O*(4∗*m*). Secondly, EWCA executes Algorithm 2, for each vertex, EWCA visits all its neighbors and it is same with Algorithm 1. Thus, the computation complexity of Algorithm 2 is *O*(2∗*m*). Thirdly, we executes Algorithm 3. We assume that EWCA obtains that the number of preliminary complex cores is |*N*(*PCC*)| according to Algorithm 2. The value of |*N*(*PCC*)| must be lower than *n*. Let us assume that the average degree is *k* in a given PPI network. Furthermore, the real PPI networks generally have sparser degree distributions and follow a power-law degree distribution [47]. Thus, *k* is generally quite small constant. For each preliminary complex core, during the expansion of a preliminary complex core, we assume that the size of the preliminary complex core *pcc*_*i*_ is |*n*(*pcc*_*i*_)|. Next, we should obtain a candidate attachment proteins subset |*Neighbor*(*pcc*_*i*_)| from the neighbor of the preliminary complex core *pcc*. The time complexity of this process is *O*(|*n*(*pcc*_*i*_)|∗*k*). After we have a candidate attachment proteins subset |*Neighbor*(*pcc*_*i*_)|, we judge whether each candidate vertex *p* should be added to the *pcc* by some conditions given in the attachment protein detection section. The time complexity of this process is *O*(|*Neighbor*(*pcc*_*i*_)|∗*k*). As a result, the time complexity of Algorithm 3 is $O\left (\sum _{i=1}^{N(PCC)}(|n(pcc_{i})|*k+|Neighbor(pcc_{i})|*k)\right)=\sum _{i=1}^{N(PCC)}k*(|n(pcc_{i})|+|Neighbor(pcc_{i})|)$. Finally, the time complexity of Algorithm 4 is *O*(|*N*(*PCC*)|). In summary, the time complexity of EWCA is $O(4*m)+O(2*m)+O\left (\sum _{i=1}^{N(PCC)}k*(|n(pcc_{i})|+|Neighbor(pcc_{i})|)\right)+O(|N(PCC)|)$.

In this paper, for the parameters selection of PEWCC, COACH and ProRank+, we use the default value according to suggestions by their authors. Similarly, because EWCA only has a structural similarity parameter, in order to ensure a fairness, we also use the default 0.4 to obtain experimental results. We run EWCA and previous clustering algorithms which have a higher degree of accuracy according to Tables 3 and 4 on two smaller PPI network datasets. In order to show that EWCA could ensure the accuracy and is also efficient. Therefore, we run them in two slightly larger PPI networks. Table 8 gives the accuracy and runtime usage of each algorithm on two species PPI networks. As Table 8 shows, experimental results show that EWCA not only has a high accuracy but also need less time than other methods. All in all, EWCA could be better balance accuracy and efficiency.

**Table 8:** Accuracy and running time by different algorithms on Human and Yeast datasets using Human complexes and Yeast complexes as standard complexes

Dataset	Algorithms	PC	F-measure	MMR	CR	Running time/s
Human	PEWCC	2930	0.3955^2*nd*^	0.0963^2*nd*^	0.5155	83.05 s ^2*nd*^
	COACH	4484	0.2455	0.0677	**0.5408** ^1 *st*^	2851 s
	ProRank+	838	0.3651	0.0687	0.2856	282.66 s
	EWCA	1979	**0.4048** ^1 *st*^	**0.0964** ^1 *st*^	0.5221^2*nd*^	**29.37 s** ^1 *st*^
Yeast	PEWCC	1353	0.3446 ^2*nd*^	0.0871 ^2*nd*^	0.4946	36.58 s ^2*nd*^
	COACH	1547	0.2083	0.0466	0.5520 ^2*nd*^	3603.31 s
	ProRank+	513	0.2712	0.0487	0.2816	251.54 s
	EWCA	924	**0.4199** ^1 *st*^	**0.0982** ^1 *st*^	**0.6182** ^1 *st*^	**18.54 s** ^1 *st*^

As the table shows, EWCA obtains best F-measure, MMR and Running time in all the two datasets. Given the results of F-measure, it shows the accuracy of protein complexes identified by EWCA is better than these comparison algorithms. The results of Running time, it is said the efficient of EWCA is faster than those algorithms. In a word, EWCA could both accuracy and efficient than some state-of-the-art algorithms with having a higher accuracy according to Tables 3 and 4. NOTE: The highest value in each row is shown in bold

### Explain the novelty of EWCA approach

Compared to earlier protein complex identification methods, EWCA possesses several advantages that are enumerated below.

1. As we all known, the reliability of existing PPIs has a great effect on the accuracy of protein complex identification methods. According to the literatures [44, 46], we define a high neighborhood-based methods based on Jaccard measure to assess the similarity of interactions.
2. The density-based methods or the core-attachment structure based methods [7, 11, 12, 15, 16] have achieved ideal performance; compared to these methods, EWCA also considers core-attachment structure and could identify protein complexes with varying densities.
3. Furthermore, EWCA has fewer parameters and provides some definitions to distinguish and identify local overlapping proteins and peripheral proteins.
4. Finally, although Wang et al. [14] consider the core-attachment structure and use the node degree and node betweenness to identify global overlapping proteins and seed proteins, then they use the modularity concept to predict overlapping protein complexes. However, it has high costs which increase with the number of nodes and edges in the PPI network and EWCA could be better balance accuracy and efficiency.

## Conclusion

In this paper, we have proposed a new method to identify protein complexes by identifying complex cores and attachment proteins. Our main contributions are as follows: (1) we define a new high-order topological similarity measure to weight each edge. (2) we further extend the protein complex cores identification methods by using the concept of structural similarity; and (3) we propose a new method to distinguish and identify local overlapping and peripheral proteins. Through the comparative analysis with other methods, the experimental results indicate that the performance of EWCA is more effective and accurate. Furthermore, each method has unique characteristics, and selecting a clustering method suitable for your purpose is important. Additionally, EWCA can balance various assessment measures, which means that EWCA provides more insight for future biological studies.

We may be able to conceive these further research directions: The available PPI data are full of noise caused by high false-positive and false-negative rates [75]. To overcome this issue, there are two ways to reconstruct a reliable PPI network by predicting new interactions among proteins [76] and designing noise-robust methods [77, 78]. In fact, methods that integrate the two strategies could enhance the performance. In addition, EWCA could be applied to cluster other biological networks, such as metabolic networks and gene regulatory networks, and it can also be used to tackle massive networks. We will further explore these applications in our future work.

## Data Availability

The datasets used and/or analysed during the current study are available from https://github.com/RongquanWang/EWCA.

## Abbreviations

CAP: Candidate attachment subset
CNS: Common neighbor support
CR: Coverage rate
EWCA: Edge Weight method and Core-Attachment structure
GO: Gene ontology
HOCN: High-Order Common neighbor
JCS: Jaccard coefficient similarity
NA: Neighborhood affinity
MMR: Maximal matching ratio
PC: Protein complex
PPI: Protein-protein interaction
SN: Structural neighborhood
SS: Structural similarity
